# Computational Analysis of Chemical Reactions Using a Variational Quantum Eigensolver Algorithm without Specifying Spin Multiplicity

**DOI:** 10.1021/acsomega.3c01875

**Published:** 2023-05-25

**Authors:** Soichi Shirai, Hokuto Iwakiri, Keita Kanno, Takahiro Horiba, Keita Omiya, Hirotoshi Hirai, Sho Koh

**Affiliations:** † Toyota Central Research and Development Laboratories, Inc., 41-1 Yokomichi, Nagakute, Aichi 480-1192, Japan; ‡ QunaSys Inc., Aqua Hakusan Building 9F, 1-13-7 Hakusan, Bunkyo, Tokyo 113-0001, Japan

## Abstract

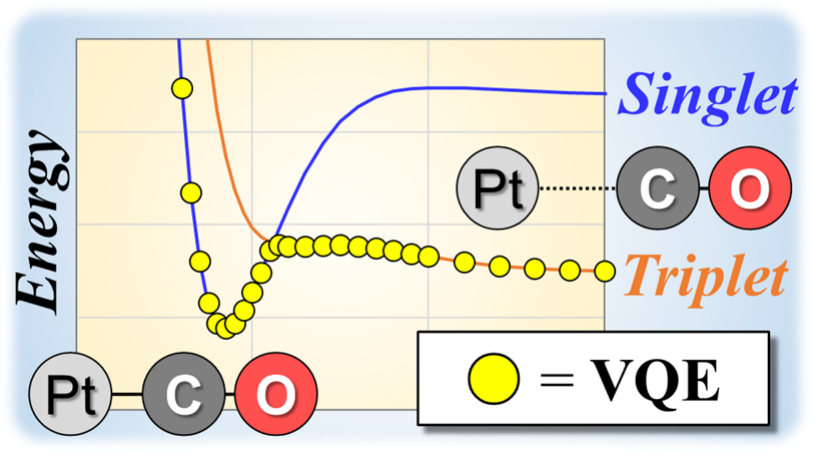

The analysis of a chemical reaction along the ground-state potential energy surface in conjunction with an unknown spin state is challenging because electronic states must be separately computed several times using different spin multiplicities to find the lowest energy state. However, in principle, the ground state could be obtained with just a single calculation using a quantum computer without specifying the spin multiplicity in advance. In the present work, ground-state potential energy curves for PtCO were calculated as a proof-of-concept using a variational quantum eigensolver (VQE) algorithm. This system exhibits a singlet–triplet crossover as a consequence of the interaction between Pt and CO. VQE calculations using a statevector simulator were found to converge to a singlet state in the bonding region, while a triplet state was obtained at the dissociation limit. Calculations performed using an actual quantum device provided potential energies within ±2 kcal/mol of the simulated energies after error mitigation techniques were adopted. The spin multiplicities in the bonding and dissociation regions could be clearly distinguished even in the case of a small number of shots. The results of this study suggest that quantum computing can be a powerful tool for the analysis of the chemical reactions of systems for which the spin multiplicity of the ground state and variations in this parameter are not known in advance.

## Introduction

Issues related to the use of fossil fuels, including the finite nature of these resources and climate change, have become significant on a global scale.^1,2^ As such, hydrogen production through water splitting and the conversion of carbon dioxide into liquid fuels have attracted much attention.^3−5^ The development of high-performance catalysts for these processes that contain reduced amounts of noble metals is also required on the basis of economics and sustainability. Quantum chemical calculations are now routinely used to simulate various reactions in conjunction with experimentation to expedite the development of such catalysts by providing an understanding of reaction mechanisms on the molecular level and to assist in material design.

The majority of current industrial catalysts utilize metal or alloy clusters, metal oxides, or metal complexes in which the metals serve as active centers.^6,7^ Accordingly, the interactions between adsorbed molecules and these metal atoms is a fundamental aspect of the catalytic reactions and must be accurately described to allow reliable simulations. However, distributing many electrons over the multiple orbitals associated with the catalytic centers and the adsorbed molecules results in numerous electronic states that are close in energy as a consequence of the degenerate or nearly degenerate d and f orbitals of the metal atoms. Describing such highly correlated electronic states requires a multiconfiguration treatment based on the configuration interaction (CI) theory^8^ instead of the more commonly used density functional theory (DFT) methods.

A CI wave function is represented as a linear combination of Slater determinants corresponding to the various electron configurations for a system. In the case of so-called full CI, all of the electron configurations generated from combinations of all of the electrons and orbitals of the system are used.^9^ Although this approach provides exact wave functions for an adopted basis set, the number of electron configurations (which, in turn, determines the computational load) rapidly increases along with the number of electrons and orbitals such that the load can easily exceed the capacity of standard computers. Consequently, only truncated CI calculations with selected electron configurations are carried out in practice, except for the simplest molecules. For these reasons, the use of quantum computers for quantum chemical calculations has recently attracted much attention.^10,11^ Quantum computers with qubits have the potential to perform full CI calculations in polynomial time by employing a quantum-phase estimation (QPE) algorithm to estimate the eigenvalues of specific Hamiltonian operators.^12−15^

In addition to advantages related to reduced computational costs, quantum chemistry calculations performed using quantum computers can also determine electronic ground states without specifying the spin multiplicity in advance. This is helpful because, in the case of certain transition metal catalysts in which the metals serve as active centers, the spin multiplicity of the electronic ground state varies depending on the molecular geometry.^16−21^ Thus, it is necessary to trace changes in the spin multiplicity along the reaction coordinates when assessing such systems. In contrast, the spin multiplicity must be specified when performing DFT calculations. For this reason, the analysis of a chemical reaction along the ground-state potential energy surface is challenging because it is necessary to compute electronic states several times while specifying different spin multiplicities and to compare the resulting energies to identify the lowest energy state. Conversely, because a superposition of multiple states having different spin multiplicities can be prepared using the qubits of a quantum computer, the ground state can, in principle, be obtained with just a single calculation employing the QPE algorithm if the initial state overlaps significantly with the ground state. As such, it will be possible to trace the reaction path along the ground state even if the spin multiplicity changes along the way without involving complicated operations, unlike the aforementioned calculations performed with conventional computers. Therefore, quantum computers are expected to serve as a useful tool for the analysis of the reactions of a system for which the spin multiplicity of the ground state is not obvious. This computational technique could also be applied to analyses of the ground states of strongly correlated systems having electronic states that are close in energy to the ground state but different in terms of spin multiplicity.^22−26^ Unfortunately, such QPE calculations require fault tolerance and cannot be executed on current quantum computers having qubits without error correction, generally referred to as noisy intermediate-scale quantum (NISQ) devices.^27−29^ Accordingly, a quantum-classical hybrid algorithm known as the variational quantum eigensolver (VQE) is widely employed for quantum chemical calculations using NISQ devices.^30,31^ These VQE calculations solve the ground-state wave function by minimizing a given cost function on the basis of the variational principle. In the case of a VQE scheme, adopting an ansatz describing multiple spin states can allow the ground state to be directly determined by minimizing the cost function.

The present study demonstrates that a reaction path along a ground-state potential energy curve including spin crossover can be traced using quantum algorithms. Specifically, the potential energy curves for the adsorption of a carbon monoxide (CO) molecule on a platinum (Pt) atom were calculated using a VQE algorithm as a proof-of-concept. This adsorption is one of the most extensively studied systems in the fields of surface and catalytic chemistry, and PtCO is the simplest model for this reaction. Prior theoretical studies have suggested that this system has a singlet ground state in its equilibrium geometry^32,33^ but a triplet state at the dissociation limit because of the triplet ground state of the neutral Pt atom.^34^ Thus, the ground state of PtCO exhibits spin crossover resulting from the interaction between the Pt and CO. It is worth mentioning that RuCO, RhCO, OsCO, and IrCO systems also exhibit a similar spin crossover in their potential energy curves.^35−37^ In this work, VQE calculations were used to calculate the ground-state potential energy curves of PtCO, including a singlet–triplet spin crossover, without specifying the spin multiplicity in advance. The spin multiplicity could be estimated efficiently on the basis of a small number of measurements using an actual quantum device even under noisy conditions, implying that the evaluation of discrete values may be a useful application of NISQ devices. The calculations without spin specification have been studied within the conventional ab initio theory for classical computers. Spin-free quantum chemistry^38−40^ has been investigated as an alternative approach to formalism based on the spin-adapted basis.^41^ The determinant-based CI calculation can provide solutions having different multiplicity by diagonalizing the CI Hamiltonian; for example, calculation with the Slater determinants with *M*_*S*_ = 0 can provide singlet and triplet solutions.^41−43^ These algorithms allow us to explore the ground state without specifying spin multiplicity in advance. Although the methods for classical computers have been well developed,^44^ calculations based on quantum algorithms are still in their early stages. This study triggers the application of quantum algorithms for such calculations.

## Theory

### VQE

The VQE method is a quantum-classical hybrid technique based on the variational principle.^30,31^ In this process, a trial wave function is constructed using a quantum computer based on the initial wave function |Ψ_o_⟩ and the ansatz quantum circuit *Û*(**θ**) with variational parameters θ. The expected value of the molecular Hamiltonian ⟨Ψ_o_|*Û*^†^(**θ**)*ĤÛ*(**θ**)|Ψ_o_⟩ is repeatedly determined with a quantum computer, while the variational parameters **θ** are repeatedly updated using a classical computer so as to minimize the cost function:
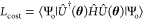
1To ensure that the computed wave function has the desired properties, a penalty term is typically included as^31,45,46^
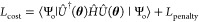
2In the case of spin multiplicity, *L*_penalty_ is typically written as
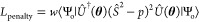
3where *w* is a weighting coefficient that determines the magnitude of the penalty, *Ŝ*^2^ is the square of the total spin angular momentum operator, and *p* is the eigenvalue of *Ŝ*^2^ that the wave function should satisfy. Note that the spin multiplicity can be specified by defining *p*. In contrast, the wave function can be calculated without specifying the spin multiplicity by setting *w* to zero. This computational scheme was utilized in the present study.

### PtCO

**Figure 1:**
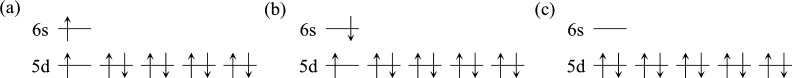
Diagrams showing the electron configurations associated with the (a) ^3^D_3_, (b) ^1^D_2_, and (c) ^1^S_0_ states of a neutral Pt atom.

**Figure 2:**
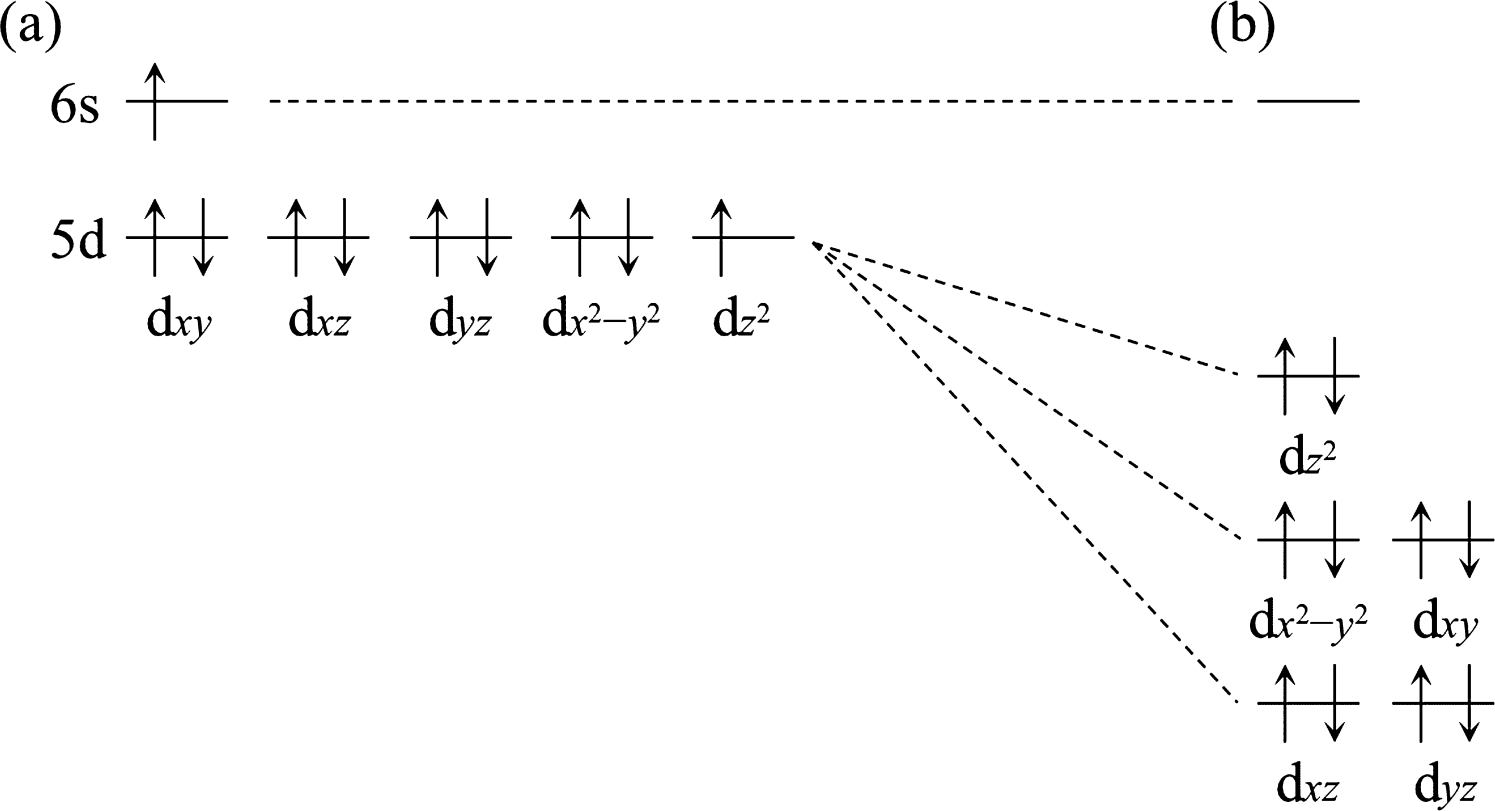
Diagrams showing the alignments of the molecular orbitals of PtCO derived from Pt 5d and 6s atomic orbitals for the (a) dissociation limit and (b) bonding region. A PtCO molecule that is linear along its *z*-axis is assumed.

This work focused on the low-lying singlet and triplet states of PtCO, either of which could be the ground state. These states could be identified on the basis of the electron configurations of the molecular orbitals derived from the Pt 5d and 6s atomic orbitals. The ground state of a neutral Pt atom is the triplet state, equivalent to the electron configuration (5d)^9^(6s)^1^, and this state is denoted herein by the symbol ^3^D_3_. The lowest energy singlet state, ^1^D_2_, has the same electron configuration as ^3^D_3_ but a total spin angular momentum of *S* = 0. In contrast, the ^1^S_0_ state is associated with the closed-shell electron configuration (5d)^10^(6s)^0^. The electron configurations of these states are illustrated in Figure 1. The energy levels of these states increase in the order of ^3^D_3_ < ^1^D_2_ < ^1^S_0_. The 6s orbital of Pt contacts and stabilizes because of the relativistic effect, which is responsible for making the ground state ^3^D_3_ with the electron configuration of (5d)^9^(6s)^1^.^47^ Thus, the ground state of PtCO at the dissociation limit is a triplet state derived from the ^3^D_3_ state of Pt, and all five d orbitals have the same energy. However, in the case of the PtCO model, the degeneracy of the 5d orbitals is removed as a result of interactions with the CO molecule. Assuming that PtCO has a linear structure, as previously suggested by a DFT study performed by Wu et al.,^33^ the Pt 5d orbitals will be split into three energy levels as shown in Figure 2. The energy gap between the Pt 6s orbital and the highest Pt 5d orbital is therefore increased by the presence of the CO. In the case that the energy gap between the Pt 5d and 6s orbitals is greater than the value for the exchange interaction, a closed-shell singlet state having a (5d)^10^(6s)^0^ configuration becomes energetically preferable to the triplet state with a (5d)^9^(6s)^1^ configuration. Therefore, the ground state of PtCO can comprise a closed-shell singlet state in the case that the Pt and CO are in close proximity to one another.

## Computational Details

Conventional quantum chemical calculations were initially performed to determine the computational procedure and conditions required for the VQE calculations. Subsequent to this, VQE calculations were performed using both a simulator and a quantum device to demonstrate that the ground state could be determined without specifying the spin multiplicity. In preparation for these calculations, the geometry of the PtCO molecular structural was optimized using the DFT method with the B3LYP functional^48−51^ together with the def2-QZVP basis set for the Pt atom^52^ and the cc-pVQZ basis set for the C and O atoms.^53^ The *Gaussian 16* program was employed for these calculations,^54^ and a linear-structured PtCO with an *r*(C–O) value of 1.1446 Å was obtained. Note that, herein, *r*(X–A) represents the interatomic distance between atoms X and A. The *r*(C–O) distance was fixed at this value in the following calculations. Plots of potential energy as a function of *r*(Pt–C) were generated over the *r*(Pt–C) range of 1.55–5.0 Å. In these calculations, the linear PtCO molecule was placed on the *z*-axis, and *C*_2*v*_ symmetry was applied. Accordingly, the symmetries of the d_*xy*_, d_*yz*_, d_*xz*_, d_*x*^2^–*y*^2^_, and d_*z*^2^_ orbitals were a_2_, b_2_, b_1_, a_1_, and a_1_, respectively, while that of the molecular orbital derived from Pt 6s was a_1_. Because a closed-shell singlet state has A_1_ symmetry, those electronic states having A_1_ symmetry were investigated in this study. It should also be noted that spin–orbit (SO) interactions were not incorporated into the calculations. SO interactions typically have significant effects on the electronic states of the systems that include heavy elements such as Pt. Considering SO coupling, the molecular symmetry should be treated in the framework of double group.^55,56^ Providing that SO coupling is incorporated, the electronic states of the PtCO system can be correctly described. Nevertheless, considering SO coupling is computationally demanding. In the present work, a qualitative description of the spin crossover was sufficient to allow the applicability of the quantum algorithm without specifying the spin multiplicity to these calculations to be ascertained. Henceforth, the electronic states of Pt treated in this study are denoted by ^3^D, ^1^D, and ^1^S. Meanwhile, we adopted a basis set with the effective core potential (ECP) for Pt,^52^ meaning that the scalar relativistic effects were considered at the ECP level.

### Calculations with Conventional Methods

The potential energy curves for PtCO were calculated using the complete active space self-consistent field (CASSCF) method^57,58^ and the complete active space configuration interaction (CASCI) method.^59^ The former approach is widely used to determine electronic states associated with multiple electron configurations. In CASSCF calculations, the molecular orbitals and the expansion coefficients of electron configurations are both optimized for the target state. Calculations at the same level of theory can be performed within the VQE framework by adopting an orbital optimization scheme (OO-VQE),^60,61^ although this requires a huge number of calculations because of the iterations involved in the VQE process, leading to a high computational cost. Therefore, the present work did not employ the orbital optimization technique. The accuracy of the CASCI calculations was also examined on the basis of a comparison with the CASSCF results. The present CASSCF and CASCI calculations were carried out using the GAMESS program.^62,63^

#### CASSCF Calculations

The accuracy of the CASSCF calculations depends on the construction of the active space, and this work employed the CAS(10e, 6o), CAS(4e, 3o), and CAS(2e, 2o) active spaces. Herein, the CAS is denoted as CAS(*M*e, *N*o) on the basis of the numbers of active electrons *M* and of active orbitals *N*. The CAS(10e, 6o) space was constructed by distributing 10 electrons over six molecular orbitals derived from Pt 5d and 6s atomic orbitals. The CAS(4e, 3o) space was constructed by distributing four electrons over three orbitals having a_1_ symmetry selected from the active orbitals of the CAS(10e, 6o) space. Finally, the CAS(2e, 2o) space was obtained by selecting the two highest energy orbitals from the active orbitals of the CAS(4e, 3o) space. The three lowest energy singlet states and the two lowest energy triplet states having A_1_ symmetry were evaluated in the calculations on the basis of the CAS(10e, 6o) and CAS(4e, 3o) active spaces. Henceforth, these states are denoted as 1^1^A_1_, 2^1^A_1_, 3^1^A_1_, 1^3^A_1_, and 2^3^A_1_. When using the CAS(2e, 2o) space, the two lowest energy singlet states and the lowest triplet state were calculated. The basis sets were def2-QZVP for the Pt atom and cc-pVQZ for the C and O atoms.

#### CASCI Calculations

The potential energy curves for PtCO associated with the two lowest energy singlet states and the lowest triplet state were calculated using the CASCI method with the CAS(2e, 2o) space. It should be noted that the results were dependent on the molecular orbitals that were considered because orbital optimization was neglected. Restricted Hartree–Fock (RHF) orbitals and restricted open-shell HF (ROHF) orbitals were both examined. The ROHF orbitals were obtained for the lowest energy triplet state, 1^3^A_1_. The effects of the basis set were also evaluated, employing the def2-SVP, def2-TZVP, and def2-QZVP basis sets for the Pt atom^52^ combined with the cc-VDZ, cc-pVTZ, and cc-pVQZ basis sets for the C and O atoms,^53^ respectively.

### VQE Calculations Using a Simulator and a Quantum Device

**Figure 3:**
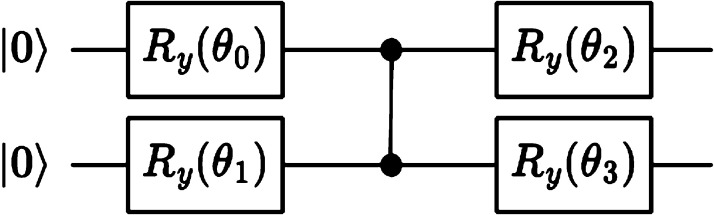
A diagram of the present ansatz consisting of *R*_*y*_ rotation gates and a *CZ* entanglement gate. The computational basis |00⟩ was employed as the initial state, and four variational parameters θ_*i*_ (*i* = 0, 1, 2, 3) for the *R*_*y*_ rotation gates were optimized during the VQE calculations.

The potential energy curves and the total spin angular momentum values for PtCO were calculated using the VQE method without penalty terms, employing both the Qiskit simulator^64^ and the IBM *ibm*_*canberra* quantum device.^65^ The basis sets used were def2-SVP for the Pt atom and cc-pVDZ for the C and O atoms. The molecular orbitals were prepared using the ROHF method implemented in PySCF.^66^ The same active orbitals together with the CAS(2e, 2o) state as used in the CASCI calculations were adopted. Parity transformation was used to map the wave function to qubits and reduce two qubits based on parity conservation of the electron number of α spin and the total electron number. As shown in Figure 3, a hardware-efficient ansatz^67^ omitting *R*_*z*_ gates with a single depth was applied to the computational basis |00⟩ to describe the target wave function in real space. The variational parameters θ_*i*_ (*i* = 0, 1, 2, 3) for the *R*_*y*_ gates, starting from random initial numbers, were optimized using the Nakanishi–Fujii–Todo (NFT) optimizer.^68^

Simulator calculations were carried out to ascertain whether the VQE algorithm itself could provide the ground state without specifying the spin multiplicity. The noise effect was ignored, and the potential energy and total spin angular momentum with statevector were calculated using conventional computers. In the case of those calculations using the *ibm*_*canberra* machine, the potential energy and total spin angular momentum of PtCO at *r*(Pt–C) values of 1.846 and 3.0 Å were determined through the Qiskit Runtime.^65^ The *r*(Pt–C) distance of 1.846 Å was chosen because the ground-state potential energy curve was found to exhibit its minimum at this point, whereas 3.0 Å was selected as a typical bond length for a triplet ground state. Three VQE calculations were performed for each molecular structure with a maximum of five iterations for the VQE optimization so as to reduce the computational cost. The Hamiltonian operator was decomposed into seven Pauli operators that were then placed into four groups using a bitwise grouping technique^31^ to reduce the number of measurements. These measurements were performed using 5008 shots for each group of Pauli operators to ascertain energies during the VQE process. The square of the total spin angular momentum operator *Ŝ*^2^, which consists of the three Pauli operators to be determined, was ascertained for the optimized state, employing 16 shots for each group. The twirled readout error extinction (T-REx) method^69^ implemented in the IBM Qiskit Runtime was adopted to reduce the readout noise effect.

## Results and Discussion

### CASSCF and CASCI Calculations

**Figure 4:**
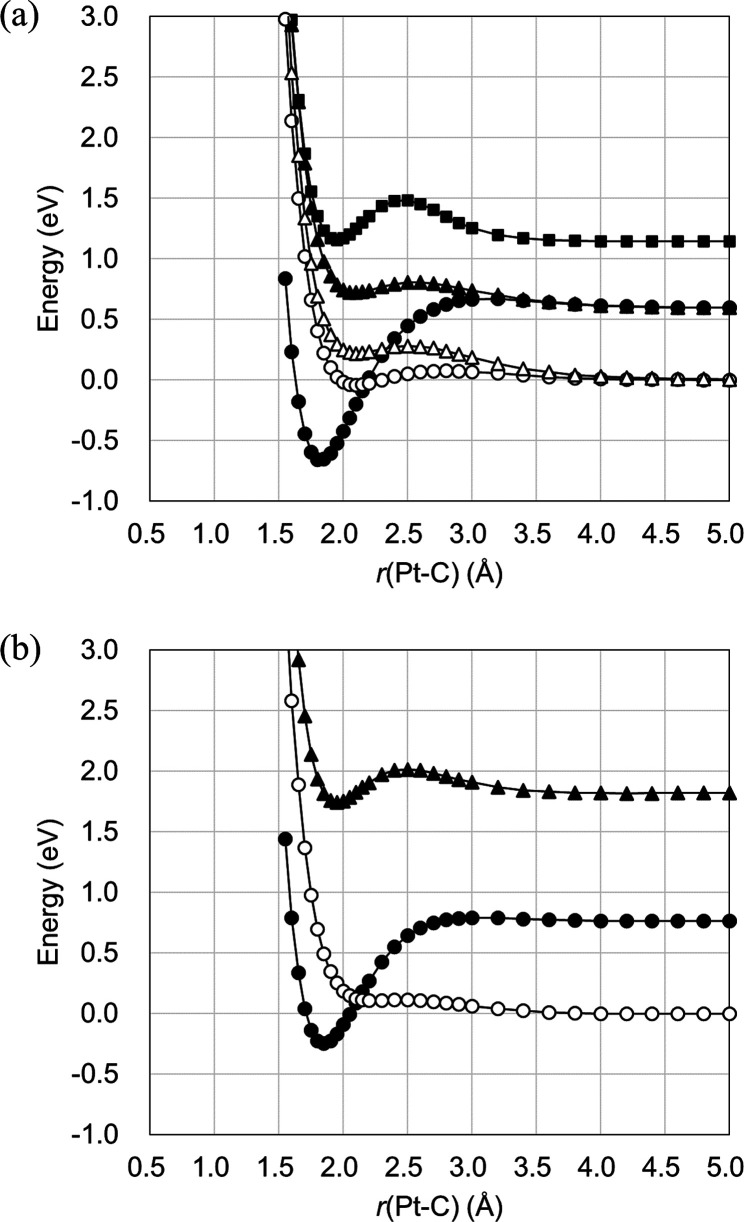
Potential energy curves for the 1^1^A_1_ (●), 2^1^A_1_ (▲), 3^1^A_1_ (■), 1^3^A_1_ (○), and 2^3^A_1_ (△) states as calculated using (a) the CASSCF method with the CAS(10e, 6o) space and (b) the CASCI method based on ROHF orbitals. The basis sets used in the CASSCF calculations were def2-QZVP for Pt and cc-pVQZ for C and O, while those in the CASCI calculations were def2-SVP for Pt and cc-pVDZ for C and O.

The total energies calculated for the electronic states are summarized in Tables S1–S5. The potential energy curves obtained using the CASSCF method with the CAS(10e, 6o) state are presented in Figure 4a. Herein, it is assumed that, at *r*(Pt–C) = 5.0 Å, the structure is at its dissociation limit. At this limit, the doubly degenerate triplet states 1^3^A_1_ and 2^3^A_1_ are the lowest energy states, while the doubly degenerate singlet states 1^1^A_1_ and 2^1^A_1_ are the second lowest, and the third singlet state 3^1^A_1_ is the highest. An analysis of the CASSCF wave function suggests that these triplet states are derived from the ^3^D state of Pt having the electron configuration (5d)^9^(6s)^1^ (Figure S1). Similarly, the 1^1^A_1_ and 2^1^A_1_ states are derived from the ^1^D state with the configuration (5d)^9^(6s)^1^, while the 3^1^A_1_ state originates from the ^1^S state with the configuration (5d)^10^(6s)^0^. The normally degenerate states are split with regard to their energy levels due to the interactions of the Pt atom with the CO. Consequently, the energetic ordering of the 1^1^A_1_ and 1^3^A_1_ states is inverted at *r*(Pt–C) = 2.17 Å, and the ground state in the region over which *r*(Pt–C) < 2.17 Å is 1^1^A_1_. Thus, the spin multiplicity of the ground state varies depending on the value of *r*(Pt–C). The potential energy curves obtained using the CASCI method based on ROHF orbitals are provided in Figure 4b. Although the energy level of the 1^3^A_1_ state is relatively underestimated here as compared to the CASSCF results, the crossover of the 1^1^A_1_ state with 1^3^A_1_ was also predicted at this level of theory and appears at *r*(Pt–C) = 2.12 Å. These results imply that VQE calculations using fewer qubits could potentially describe the spin crossover in the ground state.

**Figure 5:**
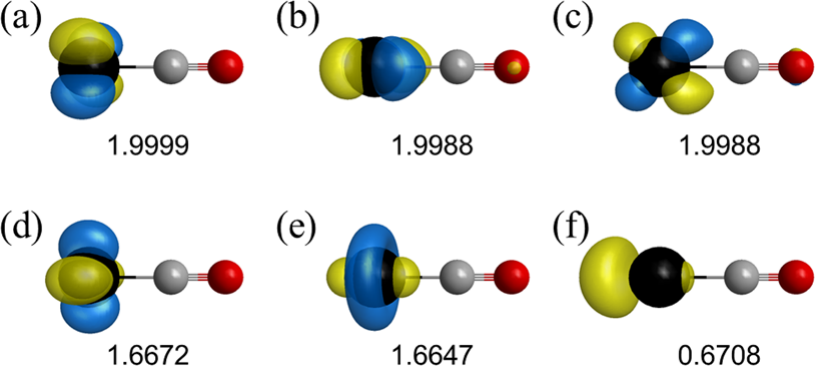
Natural orbitals at *r*(Pt–C) = 1.85 Å for the singlet states calculated using the CASSCF method with the CAS(10e, 6o) space. These orbitals are derived from the (a) 5d_*xy*_, (b) 5d_*yz*_, (c) 5d_*xz*_, (d) 5d_*x*^2^–*y*^2^_, (e) 5d_*z*^2^_, and (f) 6s atomic orbitals of Pt. The associated symmetries are a_2_, b_2_, b_1_, a_1_, a_1_, and a_1_, respectively. The occupation numbers are shown below the orbitals.

An analysis of the CASSCF wave functions with the CAS(10e, 6o) space suggested that the main configuration of the 1^3^A_1_ state was (5d)^9^(6s)^1^ with a singly occupied 5d_*z*^2^_ orbital over the entire region, while that for the 2^1^A_1_ and 2^3^A_1_ states was (5d)^9^(6s)^1^ with a singly occupied d_*x*^2^–*y*^2^_ orbital. In contrast, the weights of the main configurations for the 1^1^A_1_ and 3^1^A_1_ states varied depending on *r*(Pt–C) (Figure S1). Here, the weights were calculated as squared coefficients of the configurations in the CASSCF wave functions. The main configurations for these states were (5d)^9^(6s)^1^ with a singly occupied 5d_*z*^2^_ orbital together with the closed-shell (5d)^10^(6s)^0^ configuration. For the 1^1^A_1_ state, the configuration (5d)^9^(6s)^1^ was dominant at the dissociation limit, while (5d)^10^(6s)^0^ was dominant in the bonding region. Conversely, in the case of the 3^1^A_1_ state, the configuration (5d)^10^(6s)^0^ was dominant at the dissociation limit, but (5d)^9^(6s)^1^ was the primary configuration throughout the bonding region. The total weights of these configurations were greater than 0.85 for all states over the entire region. Figure 5 shows the CASSCF natural orbitals and the associated occupation numbers at *r*(Pt–C) = 1.85 Å on behalf of the structures in the bonding region. The sum of the occupation numbers for the orbitals with a_1_ symmetry was nearly four, while the other orbitals were approximately doubly occupied. Similar results were obtained for the dissociation limit, as shown in Figure S2. Therefore, the singlet–triplet crossover was predicted reasonably well using the smaller active spaces CAS(4e, 3o) and CAS(2e, 2o) (Figure S3).

The results of CASCI calculations based on the RHF orbitals were found to be inconsistent with those obtained using the CASSCF method (Figure S4). Specifically, the energetic ordering of the electronic states was 1^1^A_1_ < 1^3^A_1_ over the entire region, and a 1^1^A_1_–1^3^A_1_ crossover was not observed. Because the RHF orbitals were optimized for the closed-shell singlet state, the energy level of the electronic state having the primary configuration (5d)^10^(6s)^0^ could have been significantly underestimated. An analysis of the CASCI wave function revealed that one of the main configurations of 1^1^A_1_ was derived from the (5d)^10^(6s)^0^ configuration of the Pt atom, while that of the 1^3^A_1_ state originated from a (5d)^9^(6s)^1^ configuration. Thus, the energy level of the 1^1^A_1_ state was relatively underestimated, resulting in the absence of the 1^1^A_1_–1^3^A_1_ crossover. In contrast, in the case of the CASCI calculations based on ROHF orbitals, the energy level of the 1^3^A_1_ state was underestimated relative to those of the 1^1^A_1_ state because the orbitals were optimized for the lowest triplet state. Consequently, the 1^1^A_1_–1^3^A_1_ crossover was observed. The effect of the basis set was found to be minimal, and the 1^1^A_1_–1^3^A_1_ crossover could be reproduced even with smaller basis sets (Figure S5). Therefore, the combination of def2-SVP for Pt and cc-pVDZ for C and O, which were the smallest basis sets among those examined, was adopted for the VQE calculations to reduce the computational load.

### VQE Calculations

**Figure 6:**
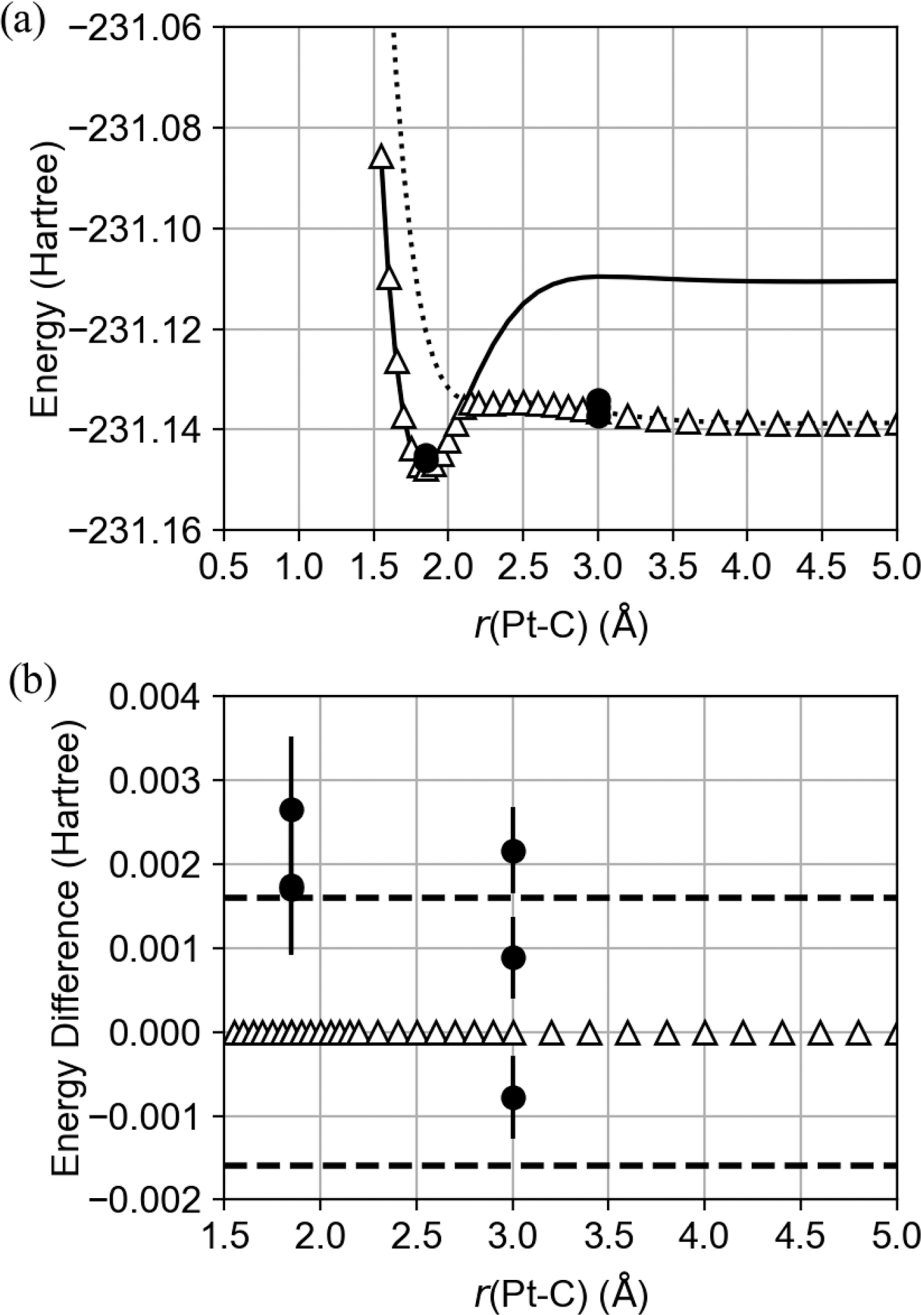
(a) Potential energy curves of the singlet ground state (solid line) and the triplet ground state (dotted line) calculated using the CASCI method, the potential energies obtained using the VQE approach with a statevector simulator (△), and the IBM *ibm*_*canberra* quantum device (●). (b) The energy difference from the CASCI potential energies obtained from the simulator (△) and the IBM *ibm*_*canberra* quantum device (●). The range indicated by the two dashed lines equals ±0.0016 hartree, equivalent to so-called chemical accuracy (±1 kcal/mol).

The potential energy curves calculated using the VQE technique with the statevector simulator are plotted in Figure 6a along with the CASCI results. These VQE calculations with the simulator reproduced the singlet potential energy curve obtained from the CASCI process over the range of *r*(Pt–C) < 2.12 Å and the triplet potential energy curve over the range of *r*(Pt–C) > 2.12 Å. Thus, the VQE values follow the ground-state potential energy curve over the entire region. These results suggest that the VQE algorithm is able to closely predict a reaction pathway along a ground-state potential energy curve including a spin crossover. The potential energies at *r*(Pt–C) = 1.846 and 3.0 Å were also calculated using the IBM quantum device, and the resulting energies and the associated errors obtained from three replicate VQE calculations are collected in Table 1. The computational time is shown in Table S6. These results qualitatively reproduce the CASCI data as plotted in Figure 6a. The calculated values were close to the singlet potential energy curve generated via the CASCI method for *r*(Pt–C) = 1.846 Å and the triplet potential energy curve obtained from the CASCI technique for *r*(Pt–C) = 3.0 Å. The differences between the potential energy values acquired from the IBM quantum device and the exact values calculated using the CASCI method are plotted in Figure 6b. It is evident from this plot that these differences were all less than ±0.003 hartree (±2 kcal/mol). The discrepancies between the actual resulting energies are larger than the standard errors of energies, suggesting the existence of a time-dependent bias that induces the displacement of measured energies during our three VQE calculations. Time-varying device noise induced during the qubit operation processes may be responsible for this bias. The discrepancy between the results obtained with the simulator and the actual quantum device can also be attributed to quantum device noise, which induces a bias that alters the measured values from the results obtained by the simulator. To mitigate the effect of noise, it is necessary to implement error mitigation techniques that not only address readout errors but also errors arising from gate operations and other sources.

**Table 1:** Potential Energies and Total Spin Angular Momentum Values Calculated Using the IBM *ibm*_*canberra* Quantum Device, Employing Three Replicate VQE Calculations^a^

*r*(Pt–C)	*E*_CASCI_	⟨*Ĥ*⟩	*e*_⟨*Ĥ*⟩_	⟨*Ŝ*^2^⟩_CASCI_	⟨*Ŝ*^2^⟩	*e*_⟨*Ŝ*^2^⟩_
1.846	–231.1477	–231.1451	0.0009	0.0	–0.06	0.19
		–231.1460	0.0008		0.01	0.19
		–231.1460	0.0008		0.36	0.19
3.0	–231.1364	–231.1342	0.0005	2.0	1.81	0.13
		–231.1372	0.0005		2.01	0.09
		–231.1355	0.0005		1.86	0.14

a *r*(Pt–C) denotes the bond length between Pt and C in angstroms, while *E*_CASCI_, ⟨*Ĥ*⟩, and *e*_⟨*Ĥ*⟩_ denote the reference potential energy calculated using the CASCI method, the expected value of the Hamiltonian, and the associated standard error, respectively, in hartree. ⟨*Ŝ*^2^⟩_CASCI_, ⟨*Ŝ*⟩^2^, and *e*_⟨*Ŝ*⟩^2^_ denote the reference CASCI value for the total spin angular momentum, the expected value for *Ŝ*^2^, and the associated standard error, respectively. The three values at each *r*(Pt–C) correspond to the results of three replicate VQE calculations.

**Figure 7:**
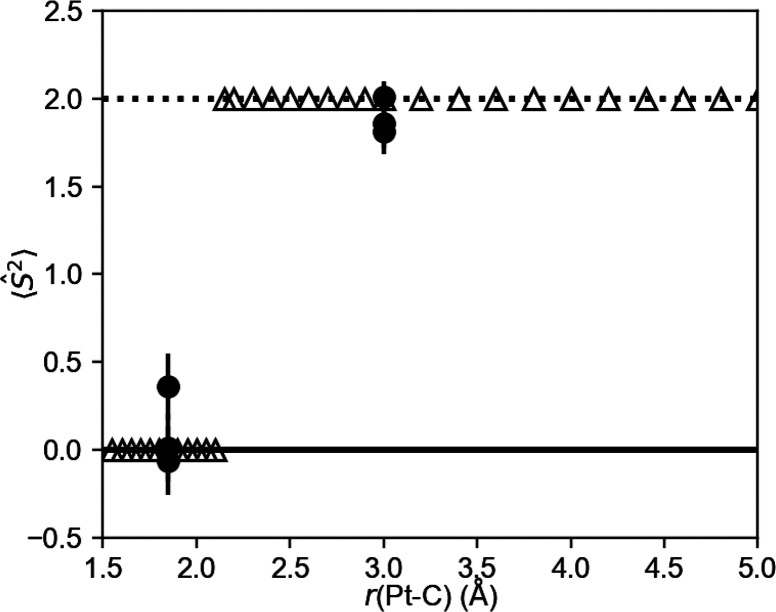
Expected values of the spin squared operator ⟨*Ŝ*^2^⟩ as obtained from the simulator (△) and the IBM *ibm*_*canberra* quantum device (●). The solid and dotted lines show the expected values of the spin squared operator ⟨*Ŝ*^2^⟩, which are 0.0 and 2.0 for the singlet and triplet states, respectively.

The calculated values for the spin squared operator, ⟨*Ŝ*^2^⟩, are also summarized in Table 1 and plotted in Figure 7. All of the calculated ⟨*Ŝ*^2^⟩ values were within ±0.5 of the reference values calculated using the CASCI technique. Consequently, the singlet and triplet states could be clearly distinguished when these values were rounded off to the nearest integers. The occurrence of a negative expectation value for *Ŝ*^2^ can be induced by both statistical error and device noise in the measurement of Pauli operators. To avoid such artifacts, it is necessary to increase the number of shots in the measurement to reduce statistical error and improve the error mitigation level to suppress device noise. The errors in these results are sufficiently small such that the change in spin multiplicity can be recognized even though only 16 shots were carried out for each Pauli term in *Ŝ*^2^. It is known that the minimal difference in the ⟨*Ŝ*^2^⟩ values between the electronic states having different spin multiplicities will be 2.0 for a system with an even number of electrons; for example, the ⟨*Ŝ*^2^⟩ values are 0.0 and 2.0 for singlet and triplet states, respectively. This value will be 3.0 for a system with an odd number of electrons; for example, the ⟨*Ŝ*^2^⟩ values are 0.75 and 3.75 for doublet and quartet states, respectively. Accordingly, the change in spin multiplicity can be detected even in the case of results that have a moderate level of accuracy when the parity of the electron number is conserved. Because the standard error associated with the expected value of the Pauli term *P̂* is , where *N* is the shot number, approximately 10 shots are sufficient to obtain the required precision in the ones place for a single Pauli term in the absence of noise. The spin squared operator used in these calculations, *Ŝ*^2^, is expressed as
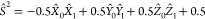
4where *X̂*_*i*_, *Ŷ*_*i*_, and *Ẑ*_*i*_ denote the Pauli operators for qubit *i* (*i* = 0, 1) along the *x*, *y*, and *z* directions, respectively. Here, we can let *e*_*P*_ equal the largest standard error among the standard errors of ⟨*X̂*_0_*X̂*_1_⟩, ⟨*Ŷ*_0_*Ŷ*_1_⟩, and ⟨*Ẑ*_0_*Ẑ*_1_⟩ using *N* shots. On the basis of the propagation of error, the standard error of ⟨*Ŝ*^2^⟩ is then
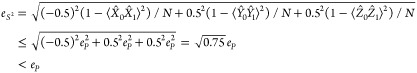
5Therefore, the use of 10 shots for a single Pauli term is sufficient to obtain the necessary numeric precision in the ones place for ⟨*Ŝ*^2^⟩ in these calculations. On this basis, the spin multiplicity of the ground state could be successfully calculated via the VQE process even with present-day noisy devices because the determination of the square of the total spin angular momentum is essentially unaffected by noise.

The total spin angular momentum *Ŝ* can be determined with a smaller number of measurements than are required for *Ĥ*. This can be explained on the basis of the number of Pauli terms that appear in the *Ŝ*^2^ operator. The spin angular momentum along the *k* (*k* = *x*,*y*,*z*) direction can be expressed as^70^
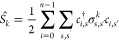
6where *n* is the number of qubits, σ_*s*,*s*′_^*k*^ denotes the (*s*, *s*′) element of the Pauli matrix in the *k* direction, and *c*_*i*,*s*_^†^ and *c*_*i*,*s*_ are the Fermionic creation and annihilation operators. The total spin squared operator *Ŝ*^2^ can be expressed as the summation of the squared spin angular momentum along the *x*, *y*, *z* directions, written as
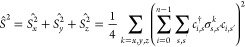
7Note that this is the product of two summations, where the index runs over all *n* qubits. Using a naive strategy, the number of Pauli terms in the observable *Ŝ*^2^ is *O*(*n*^2^), which is smaller than the number of Pauli terms *O*(*n*^4^) in the Hamiltonian *Ĥ*.

Finally, it is helpful to discuss the present limitations of the VQE algorithm as a means of determining the total spin angular momentum of the ground state. To identify the ground state among the various spin states, the ansatz should suitably represent both the ground state and other electronic states having different spin multiplicities. In addition, the number of parameters to be optimized should be sufficiently small. Unfortunately, it is still unclear whether such an ansatz exists. In this context, developing a sophisticated method for the state preparation of the quantum device will be an important challenge in future. Assuming that this is possible, the present work indicates that the total spin angular momentum could be measured with a small number of shots.

## Conclusion

Working within the framework of the VQE quantum-classical hybrid algorithm, the ground state of a molecular system was calculated while minimizing the cost function. The energy and spin multiplicity of the ground state could be simultaneously explored when applying this algorithm without penalty terms on the spin angular momentum. This computational scheme could be useful in the calculation of a strongly correlated system for which the spin multiplicity of the ground state cannot be easily estimated. In this study, the adsorption of CO onto a single Pt atom was investigated as a model system. VQE calculations using the statevector simulator successfully traced the ground state over the entire potential energy curve. These calculations converged to the singlet state in the bonding region but the triplet state at the dissociation limit. In addition, the potential energies acquired using an actual quantum device qualitatively reproduced the CASCI energy values for both the bonding and the dissociation regions. Furthermore, the spin multiplicities in the bonding and dissociation regions could be clearly distinguished despite the noise effect. Thus, using the VQE algorithm, it was possible to identify a change in spin multiplicity moving along the ground-state potential energy curve without specifying the spin multiplicity in advance. The results of this research suggest that discrete values of parameters such as spin angular momentum can be determined with reasonable accuracy even using NISQ devices if the ansatz that is adopted has sufficient expressibility to describe the multispin states, including the target state.

## Supporting Information Available

Calculated electronic state total energies (Tables S1–S5); weights of electron configurations in the CASSCF wave function with the CAS(10e, 6o) state (Figure S1); natural orbitals at *r*(Pt–C) = 5.00 Å (Figure S2); potential energy curves calculated using the CASSCF method with the CAS(4e, 3o) and CAS(2e, 2o) states (Figure S3); CASCI results based on RHF orbitals (Figure S4); and potential energy curves calculated using the CASCI method with triple-ζ and quadruple-ζ basis sets (Figure S5) (PDF)
