# Supplementary material for: Computational Analysis of Chemical Reactions Using a Variational Quantum Eigensolver Algorithm without Specifying Spin Multiplicity
Source: ACS Omega. 2023 May 25;8(22):19917–25. doi: 10.1021/acsomega.3c01875 (PMC10249088; doi:10.1021/acsomega.3c01875)
Supplement: Supplementary file 1 — ao3c01875_si_001.pdf [file ao3c01875_si_001.pdf]

Supporting Information: Computational analysis of chemical reactions using variational quantum eigensolver algorithm without specifying spin multiplicity

Soichi Shirai,[*,†] Hokuto Iwakiri,[‡] Keita Kanno,[‡] Takahiro Horiba,[†] Keita Omiya,[‡] Hirotoshi Hirai,[†] and Sho Koh[*,‡]

†*Toyota Central Research and Development Laboratories, Incorporated, 41-1 Yokomichi, Nagakute, Aichi 480-1192, Japan*

‡*QunaSys Inc., Aqua Hakusan Building 9F, 1-13-7 Hakusan, Bunkyo, Tokyo 113-0001, Japan*

E-mail: shirai@mosk.tytlabs.co.jp; koh@qunasys.com

Table S1: Electronic state total energies in Hartree calculated using CASSCF with CAS(10e, 6o). The basis sets used were def2-QZVP for Pt and cc-pVQZ for C and O. The results were plotted in Figure 4(a) in the manuscript.

r(Pt–C)	1^1A_1	2^1A_1	3^1A_1	1^3A_1	2^3A_1
1.55	-231.149596	-231.041981	-231.038449	-231.070795	-231.054113
1.60	-231.171696	-231.072580	-231.071127	-231.101627	-231.087124
1.65	-231.186850	-231.095927	-231.095278	-231.125182	-231.112250
1.70	-231.196561	-231.114448	-231.111660	-231.142908	-231.131087
1.75	-231.202061	-231.128006	-231.123048	-231.156001	-231.144947
1.80	-231.204358	-231.137683	-231.130534	-231.165448	-231.154910
1.85	-231.204279	-231.144364	-231.135027	-231.172062	-231.161859
1.90	-231.202494	-231.148773	-231.137271	-231.176508	-231.166515
1.95	-231.199546	-231.151497	-231.137879	-231.179328	-231.169461
2.00	-231.195868	-231.153003	-231.137346	-231.180955	-231.171164
2.05	-231.191804	-231.153662	-231.136072	-231.181734	-231.171991
2.10	-231.187621	-231.153758	-231.134374	-231.181931	-231.172230
2.15	-231.183516	-231.153508	-231.132505	-231.181750	-231.172098
2.20	-231.179638	-231.153071	-231.130658	-231.181342	-231.171759
2.30	-231.172901	-231.152046	-231.127585	-231.180256	-231.170894
2.40	-231.167705	-231.151200	-231.125884	-231.179197	-231.170209
2.50	-231.163838	-231.150740	-231.125753	-231.178377	-231.169932
2.60	-231.160950	-231.150707	-231.126893	-231.177843	-231.170098
2.70	-231.158801	-231.151039	-231.128741	-231.177570	-231.170641
2.80	-231.157279	-231.151624	-231.130767	-231.177506	-231.171449
2.90	-231.156301	-231.152345	-231.132634	-231.177594	-231.172406
3.00	-231.155778	-231.153113	-231.134184	-231.177785	-231.173412
3.20	-231.155680	-231.154569	-231.136256	-231.178317	-231.175300
3.40	-231.156197	-231.155759	-231.137308	-231.178869	-231.176820
3.60	-231.156818	-231.156643	-231.137810	-231.179334	-231.177935
3.80	-231.157337	-231.157264	-231.138048	-231.179681	-231.178711
4.00	-231.157713	-231.157682	-231.138161	-231.179920	-231.179234
4.20	-231.157967	-231.157955	-231.138212	-231.180075	-231.179577
4.40	-231.158131	-231.158126	-231.138235	-231.180168	-231.179800
4.60	-231.158234	-231.158233	-231.138241	-231.180220	-231.179943
4.80	-231.158300	-231.158295	-231.138241	-231.180247	-231.180035
5.00	-231.158339	-231.158333	-231.138238	-231.180258	-231.180094

Table S2: Electronic state total energies in Hartree calculated using CASSCF with CAS(4e, 3o). The basis sets used were def2-QZVP for Pt and cc-pVQZ for C and O. The results for 1^1A_1 and 1^3A_1 were plotted in Figure S3.

r(Pt–C)	1^1A_1	2^1A_1	3^1A_1	1^3A_1	2^3A_1
1.55	-231.149208	-231.042007	-231.038316	-231.070795	-231.054113
1.60	-231.171258	-231.072609	-231.070939	-231.101627	-231.087124
1.65	-231.186355	-231.095672	-231.095310	-231.125182	-231.112250
1.70	-231.195999	-231.114113	-231.111697	-231.142908	-231.131087
1.75	-231.201422	-231.127577	-231.123090	-231.156001	-231.144947
1.80	-231.203632	-231.137145	-231.130583	-231.165448	-231.154910
1.85	-231.203454	-231.143701	-231.135081	-231.172062	-231.161859
1.90	-231.201558	-231.147970	-231.137332	-231.176508	-231.166515
1.95	-231.198485	-231.150535	-231.137943	-231.179328	-231.169461
2.00	-231.194668	-231.151865	-231.137411	-231.180955	-231.171164
2.05	-231.190443	-231.152331	-231.136133	-231.181734	-231.171991
2.10	-231.186082	-231.152216	-231.134422	-231.181931	-231.172230
2.15	-231.181778	-231.151739	-231.132528	-231.181750	-231.172098
2.20	-231.177677	-231.151059	-231.130637	-231.181342	-231.171759
2.30	-231.170435	-231.149510	-231.127391	-231.180256	-231.170894
2.40	-231.164695	-231.148110	-231.125353	-231.179197	-231.170209
2.50	-231.160357	-231.147081	-231.124695	-231.178377	-231.169932
2.60	-231.157122	-231.146489	-231.125216	-231.177843	-231.170098
2.70	-231.154714	-231.146305	-231.126506	-231.177570	-231.170641
2.80	-231.152948	-231.146443	-231.128139	-231.177506	-231.171449
2.90	-231.151706	-231.146801	-231.129796	-231.177594	-231.172406
3.00	-231.150899	-231.147288	-231.131281	-231.177785	-231.173412
3.20	-231.150246	-231.148384	-231.133473	-231.178317	-231.175300
3.40	-231.150346	-231.149392	-231.134720	-231.178869	-231.176820
3.60	-231.150714	-231.150199	-231.135362	-231.179334	-231.177935
3.80	-231.151095	-231.150795	-231.135683	-231.179681	-231.178711
4.00	-231.151399	-231.151213	-231.135844	-231.179920	-231.179234
4.20	-231.151615	-231.151494	-231.135921	-231.180075	-231.179577
4.40	-231.151759	-231.151678	-231.135958	-231.180168	-231.179800
4.60	-231.151851	-231.151796	-231.135973	-231.180220	-231.179943
4.80	-231.151908	-231.151871	-231.135978	-231.180247	-231.180035
5.00	-231.151942	-231.151918	-231.135978	-231.180258	-231.180094

Table S3: Electronic state total energies in Hartree calculated using CASSCF with CAS(2e, 2o). The basis sets used were def2-QZVP for Pt and cc-pVQZ for C and O. The results for 1^1A_1 and 1^3A_1 were plotted in Figure S3.

r(Pt–C)	1^1A_1	2^1A_1	1^3A_1
1.55	-231.157046	-231.040650	-231.073862
1.60	-231.179058	-231.071036	-231.104377
1.65	-231.194118	-231.093555	-231.127693
1.70	-231.203719	-231.109793	-231.145244
1.75	-231.209082	-231.121070	-231.158213
1.80	-231.211209	-231.128481	-231.167573
1.85	-231.210920	-231.132931	-231.174127
1.90	-231.208878	-231.135166	-231.178534
1.95	-231.205622	-231.135791	-231.181326
2.00	-231.201584	-231.135297	-231.182935
2.05	-231.197107	-231.134075	-231.183701
2.10	-231.192459	-231.132431	-231.183888
2.15	-231.187849	-231.130600	-231.183700
2.20	-231.183428	-231.128762	-231.183287
2.30	-231.175565	-231.125527	-231.182196
2.40	-231.169305	-231.123317	-231.181144
2.50	-231.164555	-231.122329	-231.180346
2.60	-231.160953	-231.122475	-231.179852
2.70	-231.158154	-231.123470	-231.179633
2.80	-231.155943	-231.124959	-231.179630
2.90	-231.154198	-231.126638	-231.179780
3.00	-231.152825	-231.128314	-231.180027
3.20	-231.150911	-231.131264	-231.180643
3.40	-231.149748	-231.133495	-231.181247
3.60	-231.149040	-231.135069	-231.181743
3.80	-231.148595	-231.136144	-231.182108
4.00	-231.148300	-231.136868	-231.182359
4.20	-231.148099	-231.137348	-231.182520
4.40	-231.147954	-231.137668	-231.182619
4.60	-231.147843	-231.137883	-231.182675
4.80	-231.147760	-231.138027	-231.182704
5.00	-231.147696	-231.138125	-231.182718

Table S4: Electronic state total energies in Hartree calculated using CASCI with CAS(2e, 2o). The basis sets used were def2-SVP for Pt and cc-pVDZ for C and O. The results based on ROHF orbital are plotted in Figure 4(b) in the manuscript, while the results based on RHF orbital are plotted in Figure S4.

r(Pt–C)	ROHF orbital			RHF orbital		
	1^1A_1	2^1A_1	1^3A_1	1^1A_1	2^1A_1	1^3A_1
1.55	-231.085637	-230.974423	-231.010650	-231.102427	-230.952334	-230.973190
1.60	-231.109661	-231.007314	-231.043878	-231.127512	-230.983853	-231.003618
1.65	-231.126273	-231.031333	-231.069256	-231.144947	-231.006962	-231.026228
1.70	-231.137157	-231.048365	-231.088407	-231.156405	-231.023428	-231.042707
1.75	-231.143672	-231.059977	-231.102656	-231.163243	-231.034716	-231.054434
1.80	-231.146911	-231.067459	-231.113087	-231.166565	-231.042028	-231.062528
1.85	-231.147745	-231.071857	-231.120574	-231.167263	-231.046350	-231.067889
1.90	-231.146870	-231.074012	-231.125824	-231.166050	-231.048480	-231.071238
1.95	-231.144829	-231.074594	-231.129402	-231.163493	-231.049060	-231.073142
2.00	-231.142045	-231.074131	-231.131753	-231.160042	-231.048599	-231.074043
2.05	-231.138844	-231.073031	-231.133224	-231.156044	-231.047495	-231.074279
2.10	-231.135468	-231.071603	-231.134082	-231.151765	-231.046056	-231.074102
2.15	-231.132095	-231.070077	-231.134529	-231.147407	-231.044511	-231.073695
2.20	-231.128851	-231.068614	-231.134714	-231.143116	-231.043025	-231.073187
2.30	-231.123061	-231.066265	-231.134701	-231.135119	-231.040644	-231.072177
2.40	-231.118436	-231.064935	-231.134573	-231.128239	-231.039361	-231.071425
2.50	-231.115006	-231.064574	-231.134556	-231.122605	-231.039187	-231.071007
2.60	-231.112634	-231.064930	-231.134713	-231.118156	-231.039897	-231.070882
2.70	-231.111112	-231.065713	-231.135024	-231.114738	-231.041182	-231.070981
2.80	-231.110222	-231.066679	-231.135439	-231.112168	-231.042755	-231.071234
2.90	-231.109770	-231.067663	-231.135904	-231.110268	-231.044396	-231.071588
3.00	-231.109601	-231.068567	-231.136374	-231.108880	-231.045963	-231.071999
3.20	-231.109691	-231.069989	-231.137215	-231.107143	-231.048606	-231.072872
3.40	-231.109963	-231.070894	-231.137846	-231.106226	-231.050507	-231.073666
3.60	-231.110218	-231.071409	-231.138267	-231.105727	-231.051769	-231.074287
3.80	-231.110401	-231.071675	-231.138525	-231.105441	-231.052554	-231.074710
4.00	-231.110515	-231.071797	-231.138666	-231.105264	-231.053002	-231.074949
4.20	-231.110572	-231.071839	-231.138730	-231.105143	-231.053223	-231.075041
4.40	-231.110589	-231.071838	-231.138744	-231.105051	-231.053301	-231.075029
4.60	-231.110577	-231.071814	-231.138727	-231.104972	-231.053295	-231.074954
4.80	-231.110548	-231.071779	-231.138693	-231.104902	-231.053250	-231.074854
5.00	-231.110512	-231.071739	-231.138651	-231.104839	-231.053195	-231.074755

Table S5: Electronic state total energies in Hartree calculated using CASCI. The active space adopted was CAS(2e, 2o). The basis sets used were def2-TZVP for Pt and cc-pVTZ for C and O as "triple zeta", while def2-QZVP for Pt and cc-pVQZ for C and O as "quadruple zeta". The results are plotted in Figure S5.

r(Pt–C)	triple zeta			quadruple zeta		
	1^1A_1	2^1A_1	1^3A_1	1^1A_1	2^1A_1	1^3A_1
1.55	-231.129257	-231.022832	-231.059936	-231.142662	-231.036251	-231.073862
1.60	-231.151268	-231.053887	-231.091156	-231.163835	-231.066609	-231.104377
1.65	-231.166442	-231.076567	-231.115026	-231.178327	-231.088720	-231.127693
1.70	-231.176294	-231.092605	-231.133013	-231.187624	-231.104290	-231.145244
1.75	-231.182053	-231.103451	-231.146325	-231.192931	-231.114745	-231.158213
1.80	-231.184714	-231.110303	-231.155959	-231.195226	-231.121261	-231.167574
1.85	-231.185084	-231.114143	-231.162734	-231.195303	-231.124805	-231.174128
1.90	-231.183811	-231.115768	-231.167322	-231.193797	-231.126163	-231.178534
1.95	-231.181413	-231.115822	-231.170267	-231.191220	-231.125969	-231.181327
2.00	-231.178300	-231.114818	-231.172007	-231.187973	-231.124727	-231.182936
2.05	-231.174791	-231.113160	-231.172887	-231.184368	-231.122836	-231.183702
2.10	-231.171127	-231.111161	-231.173179	-231.180641	-231.120604	-231.183889
2.15	-231.167487	-231.109056	-231.173087	-231.176964	-231.118262	-231.183701
2.20	-231.163999	-231.107018	-231.172765	-231.173460	-231.115982	-231.183288
2.30	-231.157786	-231.103563	-231.171842	-231.167261	-231.112027	-231.182197
2.40	-231.152817	-231.101248	-231.170939	-231.162345	-231.109209	-231.181144
2.50	-231.149108	-231.100072	-231.170268	-231.158702	-231.107560	-231.180347
2.60	-231.146506	-231.099802	-231.169876	-231.156168	-231.106881	-231.179853
2.70	-231.144799	-231.100136	-231.169735	-231.154520	-231.106891	-231.179634
2.80	-231.143764	-231.100807	-231.169792	-231.153531	-231.107324	-231.179631
2.90	-231.143208	-231.101617	-231.169991	-231.153004	-231.107972	-231.179781
3.00	-231.142977	-231.102440	-231.170282	-231.152784	-231.108695	-231.180028
3.20	-231.143056	-231.103876	-231.170977	-231.152835	-231.110057	-231.180644
3.40	-231.143424	-231.104910	-231.171642	-231.153138	-231.111109	-231.181248
3.60	-231.143815	-231.105579	-231.172171	-231.153463	-231.111832	-231.181743
3.80	-231.144132	-231.105983	-231.172546	-231.153733	-231.112296	-231.182109
4.00	-231.144361	-231.106214	-231.172792	-231.153933	-231.112580	-231.182359
4.20	-231.144513	-231.106341	-231.172944	-231.154069	-231.112747	-231.182521
4.40	-231.144609	-231.106408	-231.173031	-231.154156	-231.112844	-231.182619
4.60	-231.144664	-231.106442	-231.173077	-231.154208	-231.112899	-231.182676
4.80	-231.144694	-231.106459	-231.173098	-231.154237	-231.112931	-231.182705
5.00	-231.144707	-231.106467	-231.173103	-231.154251	-231.112951	-231.182718

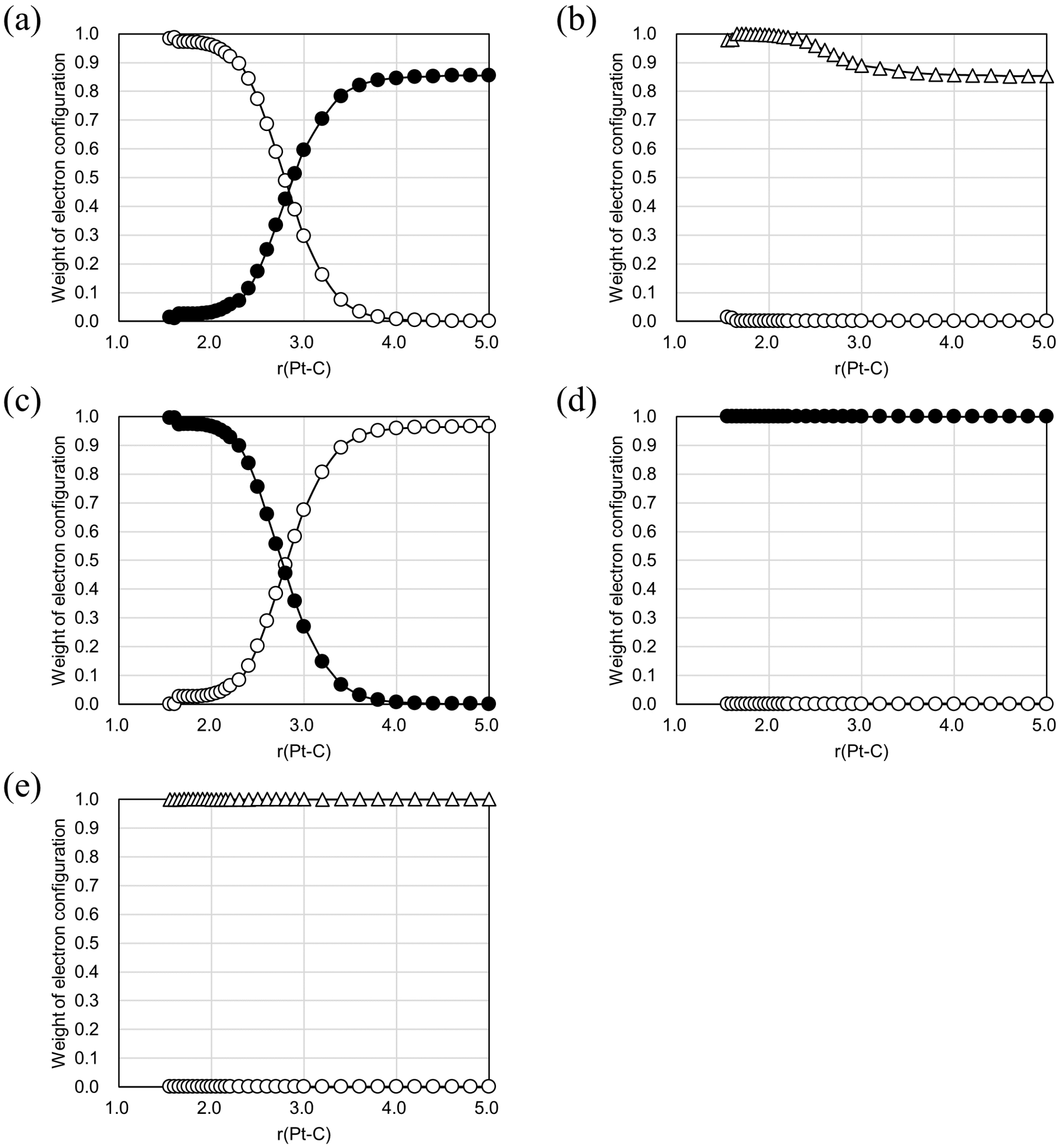

Figure S1: Weights of the electron configurations of $(5\mathrm{d}_{xy})^2(5\mathrm{d}_{yz})^2(5\mathrm{d}_{xz})^2(5\mathrm{d}_{x^2-y^2})^2(5\mathrm{d}_{z^2})^1(6\mathrm{s})^1$ (filled circle), $(5\mathrm{d}_{xy})^2(5\mathrm{d}_{yz})^2(5\mathrm{d}_{xz})^2(5\mathrm{d}_{x^2-{}^2})^2(5\mathrm{d}_{z^2})^2(6\mathrm{s})^0$ (open circle), and $(5\mathrm{d}_{xy})^2(5\mathrm{d}_{yz})^2(5\mathrm{d}_{xz})^2(5\mathrm{d}_{x^2-y^2})^1(5\mathrm{d}_{z^2})^2(6\mathrm{s})^1$ (open triangle) for (a) $1^1\mathrm{A}_1$, (b) $2^1\mathrm{A}_1$, (c) $3^1\mathrm{A}_1$, (d) $1^3\mathrm{A}_1$, and (e) $2^3\mathrm{A}_1$. The weights were calculated as squared coefficients of the configurations in the CASSCF wavefunctions with CAS(10e, 6o).

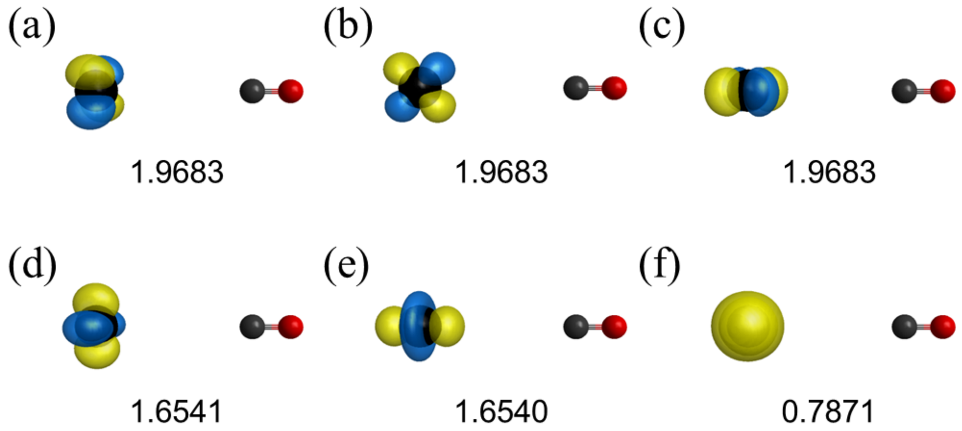

Figure S2: Natural orbitals at r(Pt–C) = 5.00 Å for the singlet states calculated using CASSCF with CAS(10e, 6o). The orbitals are derived from (a) $5d_{xy}$, (b) $5d_{yz}$, (c) $5d_{xz}$, (d) $5d_{x^2-y^2}$, (e) $5d_{z^2}$, and (f) 6s of Pt; the symmetries are a_2, b_2, b_1, a_1, a_1, and a_1, respectively. The occupation numbers are presented below the orbitals.

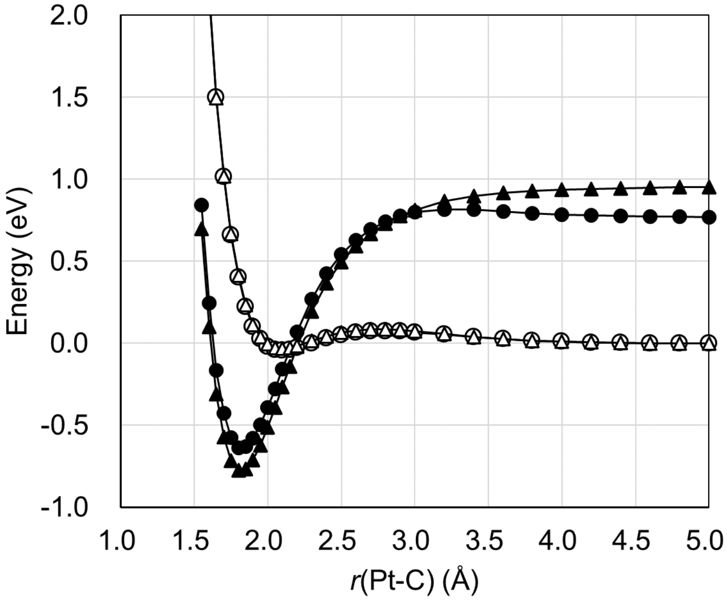

Figure S3: Potential energy curves calculated using CASSCF for 1^1A_1 with CAS(4e,3o) (filled circle), 1^3A_1 with CAS(4e,3o) (open circle), 1^1A_1 with CAS(2e,2o) (filled triangle), and 1^3A_1 with CAS(2e,2o) (open triangle). The basis set used were def2-QZVP for Pt and cc-pVQZ for C and O.

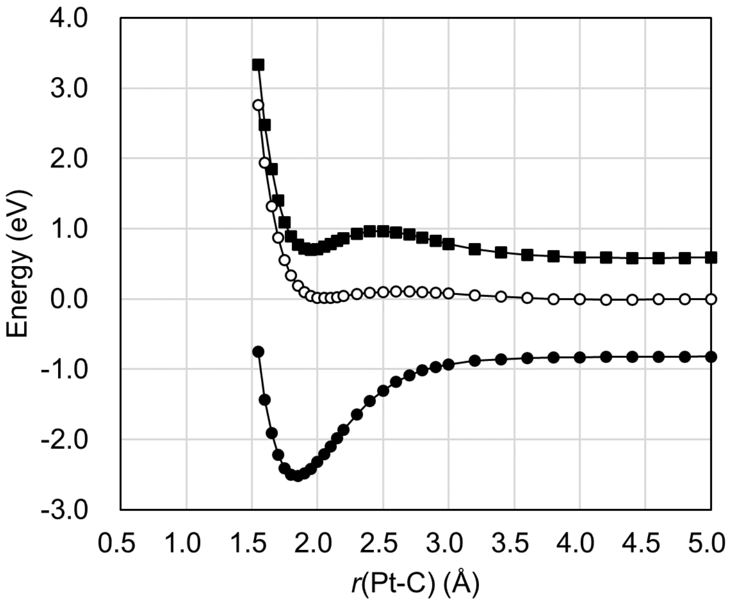

Figure S4: Potential energy curves for 1^1A_1 (filled circle), 2^1A_1 (filled square), and 1^3A_1 (open circle) calculated using CASCI with CAS(2e, 2o) based on the RHF orbitals. The basis set used were def2-SVP for Pt and cc-pVDZ for C and O.

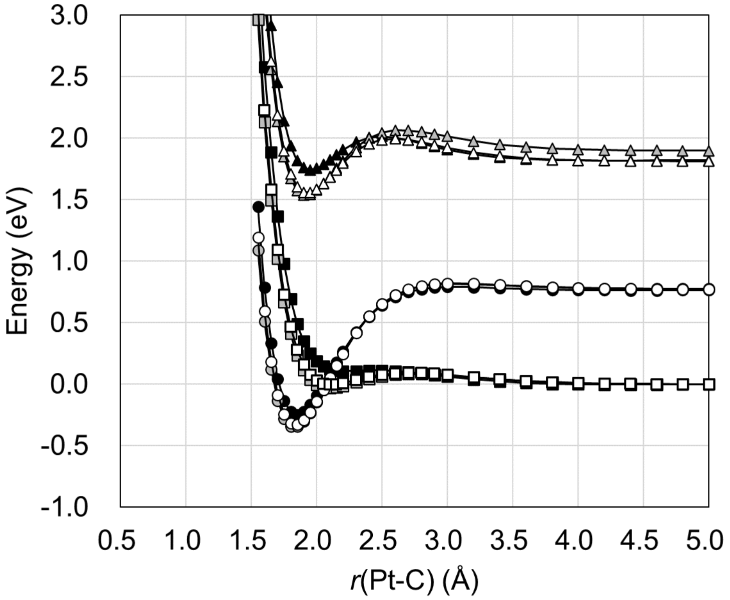

Figure S5: Potential energy curves calculated using CASCI with ROHF orbitals for 1^1A_1 (circle), 2^1A_1 (triangle), and 1^3A_1 (square); the basis sets used were def2-SVP for Pt and cc-pVDZ for C and O (filled plots), def2-TZVP for Pt and cc-pVTZ for C and O (open plots), and def2-QZVP for Pt and cc-pVQZ for C and O (gray plots).

Table S6: Computational time for the potential energies at two geometry structures of PtCO with r(Pt–C) = 1.846 Å and 3.0 Å. For the CASSCF calculations using GAMESS and the VQE calculations using the Qiskit statevector simulator, the calculations were carried out using a computer equipped with 128 GB of memory and a 2.5 GHz processor. For the VQE calculations on *ibm_canberra* device, the total time of three VQE calculations including the process on the classical computer is shown.

	Computational time
GAMESS	0.485 second
Statevector simulator	0.779 second
ibm_canberra	1 hour 50 minute
